# The atypical cell cycle regulator Spy1 suppresses differentiation of the neuroblastoma stem cell population

**DOI:** 10.18632/oncoscience.36

**Published:** 2014-05-06

**Authors:** Dorota Lubanska, Lisa A. Porter

**Affiliations:** ^1^ Department of Biological Sciences University of Windsor OntarioWindsor, ON

**Keywords:** Spdya, RINGO, Cdk, Cyclin, neurogenesis

## Abstract

Neuroblastoma is an aggressive pediatric cancer originating embryonically from the neural crest. The heterogeneity of the disease, as most solid tumors, complicates diagnosis and treatment. In neuroblastoma this heterogeneity is well represented in both primary tumours and derived cell lines and has been shown to be driven by a population of stem-like tumour initiating cells. Resolving the molecular mediators driving the division of this population of cells may indicate effective therapeutic options for neuroblastoma patients. This study has determined that the atypical cyclin-like protein Spy1, recently indicated in driving symmetric division of glioma stem cells, is a critical factor in the stem-like properties of neuroblastoma tumor initiating cell populations. Spy1 activates Cyclin Dependent Kinases (CDK) in a manner that is unique from classical cyclins. Hence this discovery may represent an important opportunity to design CDK inhibitor drugs to uniquely target subpopulations of cells within these aggressive neural tumours.

## INTRODUCTION

Neuroblastoma is the most common pediatric extracranial solid malignancy with 98% of patients diagnosed by the age of 10 [1]. The disease has an unpredictable clinical course and poor prognosis, with only 30% long-term survival [1]. Due to its origin in the migratory neural crest, neuroblastoma occurs in the peripheral nervous system arising in sympathetic ganglia and adrenal medulla [2-4]. The neural crest is a population of stem cells that derives from the neural tube during embryogenesis. Therefore, the transient embryonic structure of the tumours is reflected in the enormous genetic and phenotypic heterogeneity of neuroblastoma.

Although an inherited genetic predisposition was shown in a small subset of patients, spontaneous genetic changes are the most frequently observed. This commonly includes activation of oncogenes like MYCN and H-Ras, gain or loss of alleles, and alternations in cell ploidy [5-8]. Neuroblastoma tumours display diverse immature cell types such as neuroblasts and glial precursors and an array of heterogeneous cell types at different stages of differentiation. Accumulating *in vitro* and *in vivo* evidence suggests that alteration in cell cycle control may affect the stage of tumour differentiation and ultimately contribute to neuroblastoma pathogenesis [9]. Indeed those tumours with a poorly differentiated phenotype correlate negatively with clinical outcomes [9, 10]. In addition, self-renewing tumour initiating cells (TICs) were reported to correlate with refractory or relapse state following the initial good response to therapy observed in patients [11]. The neuroblastoma stem-like TICs were identified among the heterogeneous population in cultured human cell lines [12-14]. Interestingly, several groups demonstrated the three principle populations of neuroblastoma cells: the highly proliferative yet weakly tumourigenic N-cells, the crest derived non-neuronal S precursors and the I-type malignant and multipotent neural crest stem cells harbouring TIC potential [15]. The I-type cells express CD133 and c-kit stem cell marker proteins and are capable of self-renewal and forming rapidly growing tumours [13, 16, 17]. Importantly, the pentaspan protein CD133 was shown to regulate cell proliferation and differentiation in neuroblastoma [17]. Proliferation and differentiation are under control of the cell cycle however what regulates the balance of these decisions in the three dominant populations of cells found in neuroblastoma remains to be determined.

Spy1 (Spdya; Speedy; Spy1A; RINGO), encoded by *SPDYA* gene, is a cell cycle regulator that controls CDK2 activity and G1-S phase transition in a fashion unique from that established for the classical cyclin proteins. The Spy1-CDK2 complex does not require CDK activating kinase (CAK) -mediated phosphorylation on CDK2, and it is less sensitive to inhibitory phosphorylation by regulators such as p21^Cip1^ and p27^Kip1^ [18-21]. Thus, Spy1 is able to override several known cell cycle checkpoints, including those imposed by DNA damage [22, 23]. Both CDK2 and p27^Kip1^ play an important role in neuroblastoma progression and patient prognosis [24-26]. Notably, p27^Kip1^ accumulates in neuroblastoma cells treated with retinoids and necessitates neuronal differentiation [27], whereas increased CDK2 activity correlated with a differentiation blockage [28]. Recently we have shown Spy1 to be an important factor regulating stemness in the adult brain and human glioma [29]. Spy1 expression drove expansion of the glioma population expressing characteristic stem cell markers including CD133. Spy1 promoted clonality and suppressed multilineage differentiation potential in human glioma. Mechanistically, our data demonstrated that Spy1 is an essential driver of symmetric division of the CD133+ population in human glioma. Neuroblastoma TICs share many of the hallmark characteristics found in glioma [13, 15, 30] and the CD133+ population in neuroblastoma patient and cell line samples increases clonal expansion and tumourigenicity [30]. Hence, we sought to determine if Spy1 plays a driving role in neuroblastoma TIC populations.

This study investigated the role of Spy1 in proliferation, self-renewal and differentiation of human neuroblastoma cell lines. We find that Spy1 overexpression in the N-type neuroblastoma SH-SY5Y cells results in significantly upregulated proliferation and resistance to the 13-*cis*-Retinoic Acid (RA)-induced differentiation. Interestingly, Spy1 overexpressing cells demonstrated increased self-renewal in a neurosphere formation assay and upregulation of markers indicative of the multipotency. The forced upregulation of Spy1 levels conferred prolonged clonality and survival to serially subcultured spheres whereas downregulation of Spy1 levels resulted in decrease in tumorsphere number. Our data reveals that Spy1 is implicated in regulating CD133+ neuroblastoma cell populations. The obtained data demonstrates a role for an atypical cell cycle mechanism in driving tumourigenicity of neural crest stem cells. Importantly, this mechanism has strong implications in regulating resistance in at least subsets of neuroblastoma. Determining how to block the atypical activation of CDKs may prove a valuable therapeutic direction for neuroblastoma.

## RESULTS

### Endogenous levels of Spy1 are downregulated during RA-induced differentiation in neuroblastoma

**Figure 1:**
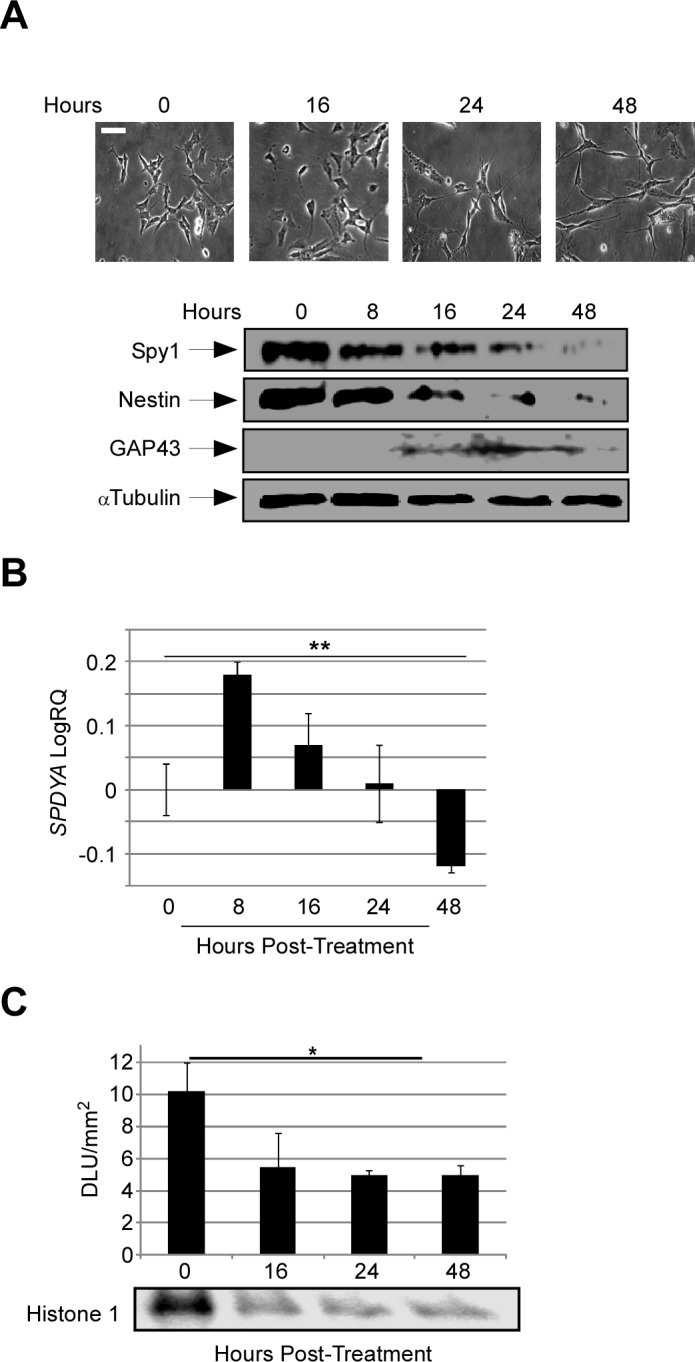
Spy1 protein levels are tightly regulated during neuroblastoma progenitor fate decisions (A) SH-SY5Y cells differentiated in 13-*cis* Retinoic Acid (RA) (2μM) and assayed at the indicated times. Differentiation was recorded by phase contrast inverted microscopy (upper panel) and cell lysates were analysed by SDS-PAGE (lower panel). Neuronal differentiation was confirmed by detection of GAP43 expression. α-Tubulin was used as a loading control. Scale bar, 50 μm. (B) *SPDYA* expression was assessed over a differentiation time course in SH-SY5Y cells by qRT-PCR. Vehicle-treated cells were used as a control. Results are presented as mean ± s.d. for triplicate samples from a representative experiment. n=4, **p < 0.01 (Student's *t*-test). (C) CDK2 activity was analyzed at the indicated time points post RA treatment in SH-SY5Y cells. Vehicle treated cells were used as a control. Phosphor-imaging analysis is demonstrated as Density Light Units (DLU) /mm2 (upper panel). Lower panel depicts a representative phosphorimage. Results are presented as mean ± s.d of a representative experiment. n=2, *p < 0.05 (Student's *t*-test).

To determine how Spy1 is expressed through differentiation in neural progenitor cells human neuroblastoma SH-SY5Y cells, were induced to differentiate over several days using RA. Cells were scored as differentiated when the neurite length exceeded twice the size of the cell body. Spy1 protein levels were abruptly depleted upon addition of RA, with levels being significantly depleted between 16-48 hours post-differentiation (Fig. 1A). This occurs concurrent with an upregulation of the differentiation marker GAP43 and a downregulation of the stemness marker Nestin (Fig. 1A; lower panel). QRT-PCR analysis upon RA stimulation revealed that endogenous *SPDYA* expression levels were significantly downregulated by 48 hours after the addition of RA (Fig. 1B). Kinase assays showed that *in vitro* CDK2 kinase activity declines in parallel with Spy1 expression levels (Fig. 1C).

### Stable overexpression of Spy1 causes delayed neural differentiation

**Figure 2:**
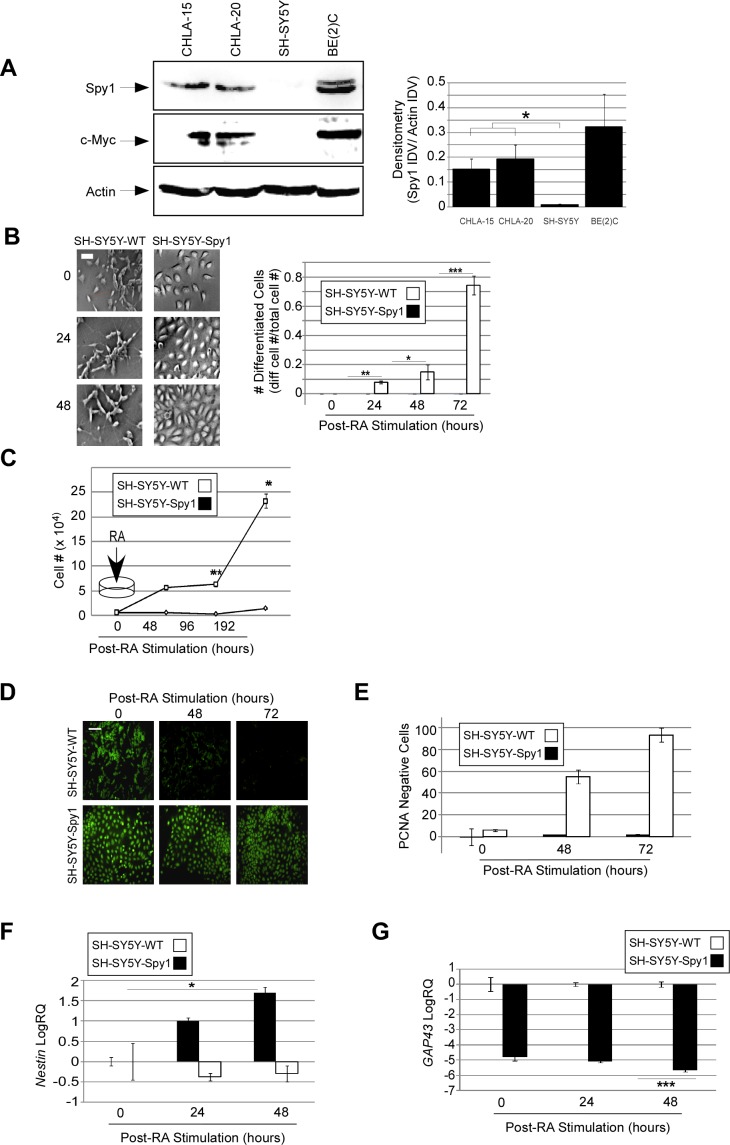
Spy1 overexpression abrogates neuronal differentiation of neuroblastoma (A) A series of neuroblastoma cell lines probed for Spy1 expression by SDS-PAGE One representative blot of 3 is depicted (left hand panel). Densitometry values are presented as the mean of Integrated Density Values (IDV) Spy1/Actin over 3 experiments (right panel). *p<0.05. (B-G) SH-SY5Y cell line was generated by stably expressing flag-tagged Spy1 (SH-SY5Y-Spy1) and empty vector (SH-SY5Y-WT). Cells were differentiated using 2μM 13-*cis*-Retinoic Acid (RA). (B) Morphology over a time course was documented using inverted phase contrast microscopy (left panel). Differentiation was scored according to axon length, and the ratio of differentiated cells to total cell number was calculated (right panel). Control bars (hollow), Spy1 overexpressing (black bars). Values are mean ± s.d. n=3; *p < 0.05, **p < 0.01, ***p < 0.001 (Student's *t*-test). Scale bar, 50 μm. (C) Cells were seeded at the density 0.5 x10^4^ cells/ml and differentiated using 2μM RA. Cells were harvested and subjected to the trypan blue analysis at indicated times. Representative data are shown as mean ± s.d. n=3, **p < 0.01, ***p < 0.001. (D) SH-SY5Y cells were subjected to BrdU incorporation along the RA-induced differentiation time course. Immunocytochemistry was conducted at 48 hours and 72 hours. PI was used as a nuclear counterstain (not shown). Scale bar, 100μm. (E) PCNA negative cells and total cell number were scored in 3 different fields of view at 48hours and 72hours time points. PCNA negative cells are expressed as a percentage of total cell number at each time point. Data are shown as mean ± s.d; n=2. (F & G) Expression levels of (F) *Nestin* and (G) *GAP43* were analyzed by qRT-PCR in SHSY5Y-WT (WT) and SH-SY5Y-Spy1 (Spy1) along differentiation time-course. Representative data are shown as mean ± s.d. n=3; *p < 0.05 (E) and ***p < 0.001 (F).

We tested a panel of neuroblastoma cell lines for Spy1 protein expression levels (Fig. 2A). It was noted that SH-SY5Y cells had significantly lower levels of Spy1 than levels found in CHLA15, CHLA20 or BE(2)C cells. This correlated with protein levels of the oncogene c-Myc, known to transcriptionally upregulate Spy1 levels [32]. To determine the effect of abnormal elevation of Spy1 protein on differentiation in neuroblastoma, Spy1 or an empty vector control were stably overexpressed in the SH-SY5Y cells. SH-SY5Y-WT (expressing empty vector) and SH-SY5Y-Spy1 (Spy1 overexpressing) cell lines were induced to differentiate and observed over a 72 hour time course. By 72 hours post-differentiation over 75% of control cells successfully differentiated while no signs of differentiation were visible in the Spy1 overexpressing cells (Fig. 2B; right panel). To determine whether effects could be due to the known proliferative effects of Spy1, cell numbers, BrdU incorporation and PCNA staining was conducted (Fig. 2D & 2E). At 96 hours post-differentiation SH-SY5Y-Spy1 cells continued to proliferate, while SH-SY5Y-WT cells had significantly reduced proliferation, indicative of terminal differentiation (Fig. 2C). This was also demonstrated by incorporation of BrdU (Fig. 2D) and PCNA (Fig. 2E). In each assay Spy1 overexpressing cells continued to synthesize DNA and cycle at 72 hours post-differentiation while over 50% of control cells enter quiescence by 48 hours post-differentiation. Interestingly, qRT-PCR of cells overexpressing Spy1 in the presence of differentiation stimuli revealed significantly higher levels of the neural stem cell (NSC) marker *Nestin* (Fig. 2F) and significantly lower expression of the neuronal differentiation marker, growth associated protein 43 (*GAP43*) (Fig. 2G), than control counterparts.

### Spy1 regulates self-renewal in neuroblastoma cells

**Figure 3:**
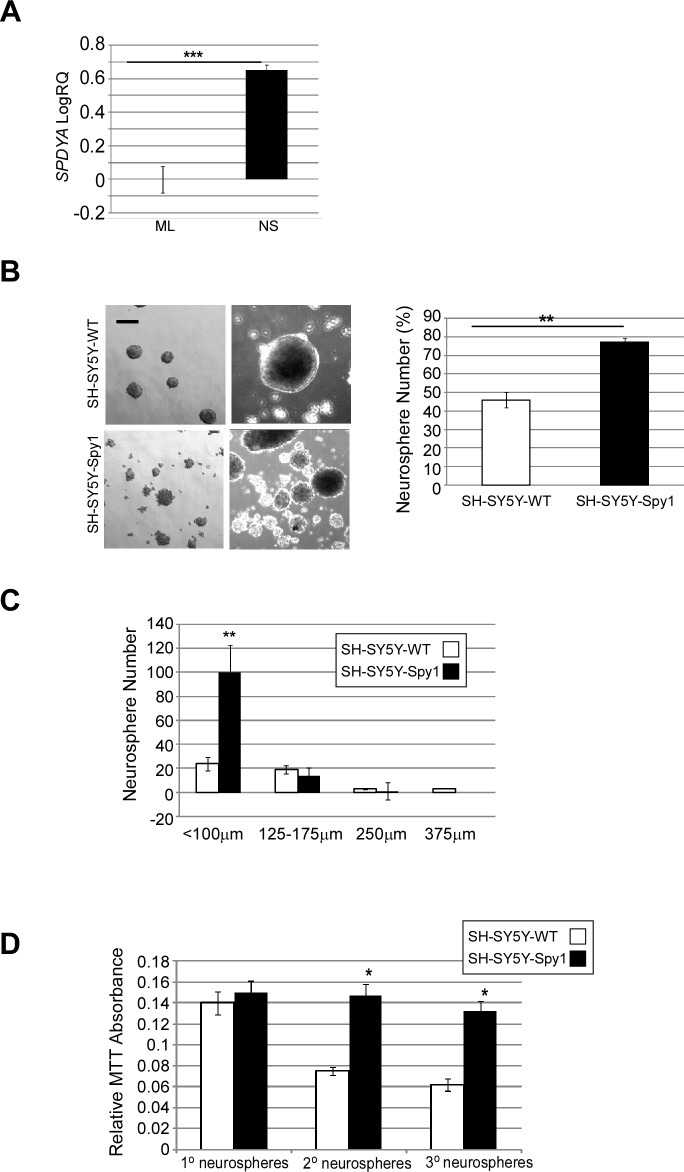
Elevated levels of Spy1 protein promote neuroblastoma progenitor self renewal (A) SH-SY5Y cells cultured as neurospheres (NS) or in monolayer (ML) were harvested and *SPDYA* expression levels determined by qRT-PCR. Data shown is mean ±s.d, ***p < 0.001. (Student's *t*-test, n=3). (B-D) SH-SY5Y-WT and SH-SY5Y-Spy1 cells were grown in neurosphere assays. (B) Morphology of cultures by light microscopy (left panels). Scale bar, 100 μm. Spheres were passaged every 6-7 days and the neurosphere formation efficiency was quantified as a number of spheres relative to the total number of the cells seeded (right panel). Representative data are shown as mean ±s.d. of triplicates from three independent experiments, **p < 0.01 (Student's *t*-test; n=3). (C) Neurospheres maintained in culture for 14 days were scored according to diameter. Values presented as mean ±s.d. of two independent experiments, **p < 0.01. (Student's *t*-test). (D) Primary, secondary and tertiary neurospheres were subjected to MTT assay. The obtained absorbance at 590nm was corrected for background absorbance. Data shown is mean ±s.d, *p < 0.05 (Student's *t*-test; n=2).

The pool of multipotent NSCs can be purified from heterogeneous cell population utilizing a neurosphere formation assay. Unlike neural progenitor cells with limited proliferative potential, only highly proliferative multipotent NSCs can retain the ability to self-renew and produce neurospheres that can be passaged in a long term culture [33]. Additionally, neurospheres derived through this assay retain the capacity to express GAP43 and glial fibrillary acidic protein (GFAP) upon differentiation conditions [34]. SH-SY5Y neuroblastoma cells have been previously shown to express markers of typical neural crest stem cells [35, 36], and to contain populations of self-renewing, multipotent tumour cells [30, 37]. Interestingly, we found that endogenous levels of Spy1 were significantly elevated in cells cultured as neurospheres when compared to monolayer culture, supporting the endogenous requirement for this protein in maintaining multipotency (Fig. 3A). We investigated the influence of Spy1 overexpression on self-renewal in SH-SY5Y cells cultured as neurospheres. Spy1 overexpressing cells almost doubled the number of neurospheres as compared to controls (Fig. 3B). Increases in neurosphere number were significant for neurospheres of a diameter smaller than 100μm; however, there was no significant increases seen for neurospheres larger than 100μm (Fig. 3C), notably cultures were carried out in a flat dish and not in aggregate culture [38]. Serial passaging of neurospheres demonstrated that Spy1 overexpressing populations had enhanced longevity over control neurospheres (Fig. 3D).

### Spy1 regulation of stemness and neural lineage commitment is controlled by extracellular microenvironment

**Figure 4:**
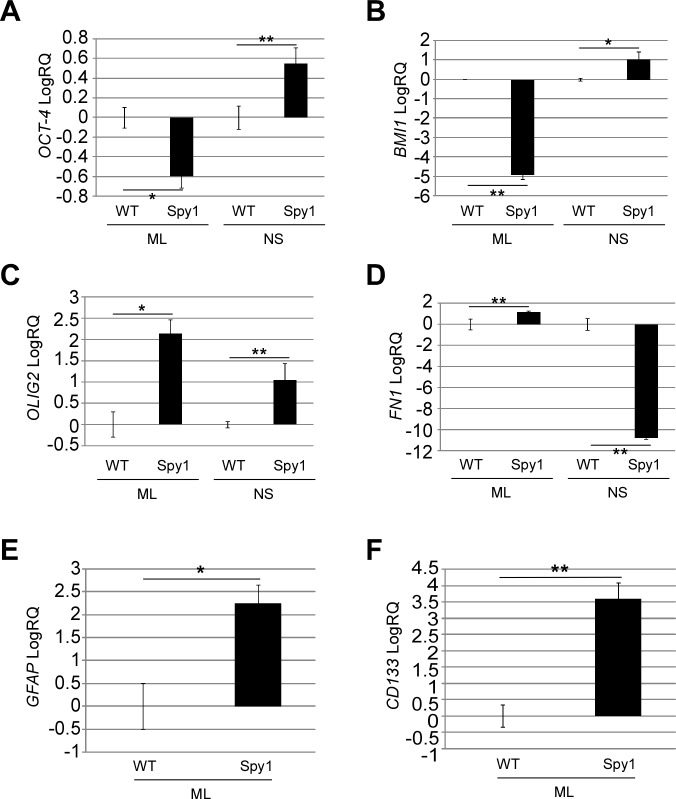
Spy1 regulation of stemness and neural lineage commitment is controlled by extracellular microenvironment (A-F) SH-SY5Y control cells (WT) or overexpressing Spy1 (Spy1), cultured in monolayer (ML) or as neurospheres (NS). mRNA levels were analysed using qRT-PCR. Expression levels of (A) *OCT-4*, (B) *BMI1*, (C) *OLIG2*, (D) *FN1*, (E) *GFAP*, and (F) *CD133*. Representative data are shown as mean ±s.d. of triplicates from three independent experiments, *p < 0.05, **p < 0.01 (Student's *t*-test).

Given the environmental differences seen with Spy1 expression in neurosphere culture, corresponding passages of SH-SY5Y-WT or SH-SY5Y-Spy1 cells were cultured either in monolayer or as neurospheres and gene expression was analyzed. Expression of the pluripotency marker *Oct-4* (Fig. 4A) and the stem cell marker *BMI1* (Fig. 4B) were significantly elevated by Spy1 when cultured as neurospheres but not in monolayer culture. Spy1 expression significantly elevated the expression of glial progenitors and stem-like TIC markers (Fig. 4C-F). Specifically, Spy1 enhanced expression of the gene for the glial progenitor marker *OLIG2* (Fig. 4C), the astrocyte-specific marker *GFAP* (Fig. 4E) and the TIC marker *CD133* (Fig. 4F). Spy1 also enhanced expression of the gene for fibronectin (*FN1*), which has been shown to mediate invasiveness and cell survival in other types of neural derived tumours (Fig. 4D) [39-41].

### Depletion of Spy1 in neuroblastoma decreases cell proliferation and clonality

**Figure 5:**
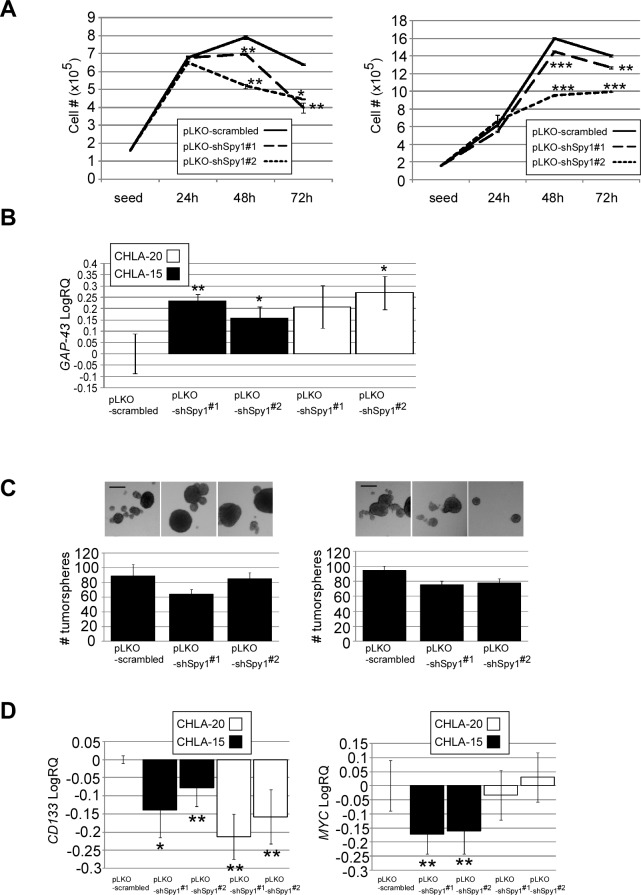
Depletion of Spy1 in neuroblastoma decreases cell proliferation and clonality (A) Proliferation kinetics of CHLA-15 (left) and CHLA-20 (right) cells infected with two shRNA constructs against Spy1 (pLKO-shSpy1#1, pLKO-shSpy1#2) or scrambled control (pLKO-scrambled). (B) *GAP-43* expression in pLKO-shSpy1#1, pLKO-shSpy1#2 or pLKO-scrambled CHLA-15 and CHLA-20 cells. (C) Number of neuroblastoma tumorspheres formed by CHLA-15 (left) or CHLA-20 (right) cells infected with pLKO-shSpy1#1, pLKO-shSpy1#2 or pLKO-scrambled. (D) *CD133* (left) and *c-MYC* (right) expression in pLKO-shSpy1#1, pLKO-shSpy1#2 or pLKO-scrambled CHLA-15 and CHLA-20. (A-D) Data is expressed as mean ±s.d. of triplicates from at least three independent experiments, *p < 0.05, **p < 0.01, ***p<0.001 (Student's *t*-test).

Given the heterogeneity of tumours and reported populations of cancer stem cells in neuroblastoma we addressed the role of Spy1 in stemness properties of cell lines characterized by higher Spy1 protein (Fig. 2A). Spy1 levels were depleted in heterogeneous populations of the CHLA-20 and CHLA-15, both shown to contain high endogenous levels of Spy1 (Fig. 2A). CHLA-20 line contains a pool of CD133+/CD34+/Nestin+ TICs [30] and both CHLA-20 and CHLA-15 cell lines are resistant to conventional chemotherapy [42]. Spy1 knock-down using two different shRNA constructs caused significant decrease of CHLA-15 and CHLA-20 cell proliferation (Fig. 5A, left & right panel respectively) and increase in the levels of GAP-43 neuronal marker mRNA levels (Fig. 5B). To determine the role of Spy1 in self-renewal of these cells we subjected Spy1 knockdown cells to neurosphere formation assay. A decrease in the number of spheres formed in both cell lines was noted, although this was not significant (Fig. 5C, left & right panel). The downregulation of Spy1 was followed by a significant decrease in CD133 in both CHLA-15 and CHLA-20 cell lines, and a significant decline in c-MYC mRNA levels in CHLA-15 cells (Fig. 5D). These results demonstrate that Spy1 downregulation is required for neuronal differentiation to take place in heterogeneous populations of neuroblastoma cells and support that Spy1 is required for maintaining proliferation and aspects of stemness characteristics in neuroblastoma TICs.

### Clonal properties of CD133+ populations of neuroblastoma depend on Spy1

**Figure 6:**
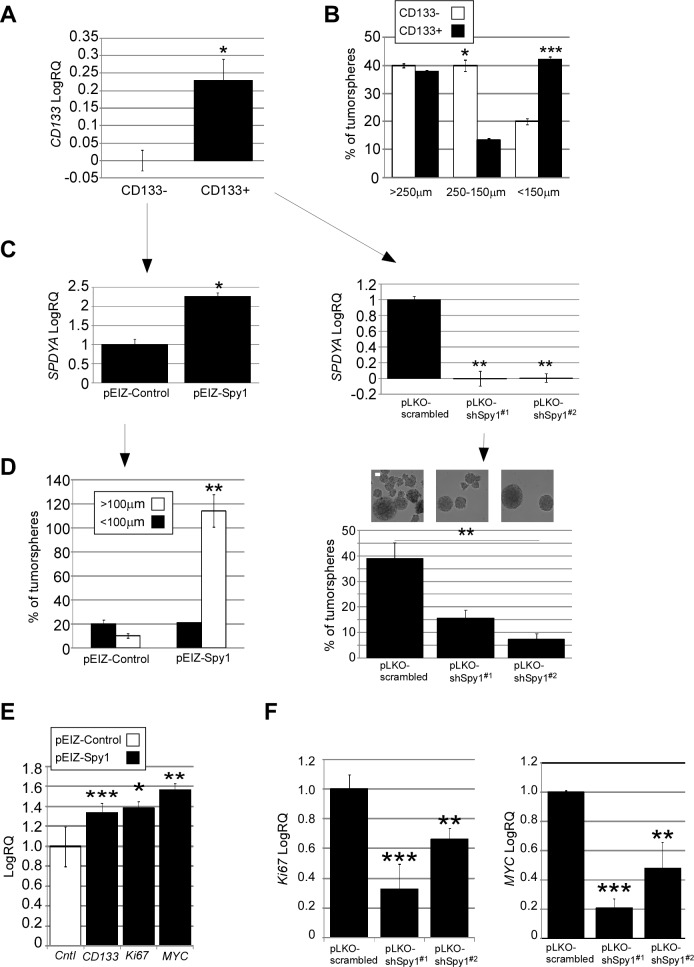
Clonal properties of CD133+ populations of neuroblastoma depend on Spy1 (A) CHLA-15 cells sorted for CD133. Expression levels of CD133 depicted in CD133+ vs. CD133- population. (B) CD133- and CD133+ populations analyzed for the number and size of spheres formed. (C; left) Spy1 mRNA levels in CD133- cells subjected to Spy1 overexpression. (C; right) CD133+ population subjected to knockdown control (pLKO-scrambled) or Spy1 knockdown (pLKO-shSpy1 #1 and #2). (D) Number and size of tumorspheres formed in CD133- cells overexpressing Spy1 (left) or CD133+ Spy1 knockdown cells (right). (E) CD133- population overexpressing Spy1 assessed for expression of *CD133*, *Ki67* or *c-MYC*. All genes are expressed relative to *GAPDH* and pEIZ-Control(cntl) genes are normalized to 1. pEIZ-Spy1 expression is shown relative to pEIZ-Control. (F) *Ki67* (left) *and MYC* (right) expression in CD133+ cells expressing pLKO-shSpy1#1, pLKO-shSpy1#2 or pLKO-scrambled. (A-F) Data is expressed as mean ±s.d. of triplicates from three independent experiments, *p < 0.05, **p < 0.01, ***p<0.001 (Student's *t*-test).

To test the hypothesis that Spy1 is involved in regulating the CD133+ subpopulation within neuroblastoma, as previously determined in glioma [29], we sorted CHLA-15 cells for CD133 and subjected negative and positive populations to neurosphere formation assays (Fig. 6A). We found no significant difference in the overall number of the spheres formed between CD133+ and CD133- populations (data not shown), however CD133+ populations formed significantly more spheres of a smaller size, while CD133- populations formed larger spheres (Fig. 6B). When Spy1 expression was driven in the CD133- population more spheres, particularly of the smaller size, were formed (Fig. 6C, left panel). Spy1 overexpression in this population correlated with an increase in the levels of CD133, c-MYC and Ki67, TIC and proliferation marker levels in CD133- cells (Fig. 6E). In opposition to this, Spy1 knockdown in CD133+ cells (Fig. 6C, right panel) resulted in significant decline in the number of spheres formed (Fig. 6D; right panel) and decreased expression levels of *Ki67* and c-MYC (Fig.6F). We were not able to amplify *CD133* in Spy1 knockdown CD133+ samples (not shown).

Overall, our results revealed that Spy1 is a necessary driver of proliferation and stem-like characteristics in neuroblastoma, particularly in the CD133+ TIC population. Elevated levels of Spy1 in neuroblastoma populations suppresses differentiation and promotes TIC characteristics.

## DISCUSSION

Inappropriate differentiation of immature cells within the ganglionic lineage is thought to drive neuroblastoma [2-4]. Indeed, the heterogeneous populations of diverse progenitor cells, seen to exist within the tumour mirror, neural crest plasticity [12-14]. Multiple studies over the years have attempted to elucidate the molecular basis behind the differentiation processes of neuroblastoma. Diverse agents, growth factors and differentiation protocols have been proposed [43-46]; however, the information about the role of the cell cycle control in neuroblastoma remains insufficient. We report for the first time that an atypical G1 phase regulator, Spy1, is implicated in differentiation and self-renewal of neuroblastoma cells.

We utilized the SH-SY5Y cell line of an established N-cell phenotype that is blocked at the precursor stage of neuronal development. We find that endogenous Spy1 levels are reduced during cell differentiation and that preventing this downregulation also prevents RA-induced differentiation. Overexpression of Spy1 resulted in enhanced self-renewal and longevity of neuroblastoma cells cultured in a neurosphere formation assay. Spy1 levels were also significantly elevated in self-renewing neurosphere populations, as compared to cells cultured as a monolayer. Collectively, this suggests a potential role of the Spy1 protein as a stabilizer of proliferation among cell populations of higher hierarchy, namely progenitors and TICs. Spy1 has demonstrated roles in spinal cord regeneration [47], it was found to possess stem-like qualities in the developing mammary gland [32] and in the subventricular zone stem cells in the adult brain [29], supporting a general role for Spy1 in select populations of adult stem or progenitor cells.

Spy1 is a direct activator of CDK2 [48], and Spy1 can indirectly activate CDK2 by promoting the degradation of the CDK inhibitor p27^Kip1^ [20]. Dobashi *et. al.* demonstrated that a decrease in CDK2 kinase activity must occur in order to support functional differentiation [49] and others have demonstrated that p27^Kip1^ levels accumulate during neuronal cell differentiation [50]. Interestingly, suppression of CDK2 activity is required for reduced proliferation and survival of the primary neural crest derived tumour and its inhibition was demonstrated to be synthetic lethal in MYCN overexpressing neuroblastoma [26, 51]. Spy1 direct interactions with p27^Kip1^ promote the phosphorylation on Thr-187 of p27^Kip1^, a modification that is required for its ubiquitination by SCF^Skp2^complex and its subsequent proteolysis [52-54]. High expression of the Skp2 component of the SCF^Skp2^ ligase was included in the genetic signature of aggressive stages of neuroblastoma. Skp2 overexpression correlated with p27^Kip1^ protein downregulation and poor patient prognosis [55, 56]. Hence, Spy1-mediated CDK2 activity may affect the outcome of patients with Skp2 overexpression.

We observed a differential effect of Spy1 on neuroblastoma cells when grown in neurosphere culture, versus the general population of cells grown in monolayer culture. In neurosphere culture Spy1-induced expression of the progenitor and multipotency markers BMI11 and OCT-4, respectively. Both BMI1 and OCT-4 were shown to exert their functions in the other neural tumours by preventing cellular differentiation and contributing to their growth and progression [57, 58]. Hence, this data supports the hypothesis that Spy1 may function in promoting the growth of highly undifferentiated cells in neuroblastoma. When cells were in monolayer, however, Spy1 promoted expression of glial progenitors such as *GFAP* and *OLIG2*. SH-SY5Y cells were demonstrated previously to contain a minute pool of highly clonogenic S-cells [59] that are also characterized by expression of glial markers [13]. Thus, forced expression of Spy1 can be speculated to contribute to the expansion of self-renewing cell populations within the heterogeneous tumour. We show for the first time that the proliferation and clonal expansion of CD133+ neuroblastoma TICs relies on Spy1, and that Spy1 overexpression increases while its knockdown leads to decrease in *c-MYC* expression levels in this population. Consistently our results show that c-myc protein levels correlate with Spy1 expression in all neuroblastoma cell lines tested. We have reported previously that Spy1 expression is regulated downstream of c-myc [32]. Whether c-myc regulation takes place in neuroblastoma requires further investigation. These data support a potential role for Spy1 in maintaining stem-like TIC population in neuroblastoma. Careful analysis is required to determine if Spy1 manipulation affects specific subpopulations differently.

## METHODS

### Cell lines

Using BBS/CaCl_2_ delivery (pH 7.05, 37°C and 3% CO2) PT67 packaging cells were transiently transfected with the DNA construct of choice. Following an 8 hour incubation (37°C and 3% CO2) media was changed, and cells were allowed to recover at 37°C and 5% CO_2_. 24 hours later cell media containing the virus were harvested, centrifuged for 5 minutes at 2,000 g and filter sterilized using a 0.45 μm filter. An overnight amplification (P2) was also collected, filter sterilized and stored at −80°C. Cells were infected with P2 virus at ~70-80% confluency and incubated overnight in 1:2 volume/volume virus to cell media ratio containing polybrene (25 μg/ml). Media was changed the next day and 24 hours later infected cells were selected in media containing 600 μg of G418. Individual colonies, as well as mixed populations of cells, were maintained and the incorporation of cDNA was checked using genomic DNA and primers spanning the exon-exon junctions. SH-SY5Y cells were obtained from ATCC. Stable SH-SY5Y lines were selected and cells were maintained in media containing 200 μg/mL of G418. CHLA-15 and CHLA-20 cell lines were obtained from Children Oncology Group repository (University of Texas). Infected cells were selected with 1ug/ml of Puromycin.

### Differentiation assays

SH-SY5Y cells were grown on 60 mm or 100 mm plates to 60% confluency. 13-*cis*- Retinoic Acid (2 μM) was added to SH-SY5Y growth media to induce neurite outgrowth. Cover-slips and cell pellets were harvested each day, and control samples (kept in growth media) were harvested on day 1. Cells were pelleted by centrifugation at 13,000 rpm for 15 minutes at 4°C, supernatant was removed, and pellets were stored at –20°C until use. Cell pellets were lysed in 0.1% NP-40 lysis buffer (0.1% NP-40, 20 mM Tris pH 7.5, 5 mM EDTA pH 8.0, 100 mM sodium chloride) for 1 hour, with mixing every 10 minutes. Samples were centrifuged again at 13,000 rpm for 15 minutes at 4°C to remove cell debris, and stored at –20°C until use. Imaging was done on the AxioSkope2 Plus microscope (Zeiss) using Northern Eclipse computer software.

### BrdU assay and fluorescent detection

SH-SY5Y cells were grown on cover slips in 60 mm culture dishes. Over a differentiation time course BrdU (Cat# 550891; BD Sciences) was added to the media to a final concentration of 10 μM, incubated for 30 minutes and then cover slips were harvested. The cell culture density never exceeded 2×10^6^ cells/ml. Cells were fixed with 70% ethanol for 30 minutes at room temperature followed by incubation with 0.07N NaOH for 2 minutes and neutralization in PBS, pH 8,5. Primary antibody against BrdU (Cat# 347580, BD Sciences) was applied for 30 minutes in humidified chamber. The cells were washed 3 times with PBS and incubated with secondary antibody conjugated to Alexa-488 fluorophore (A11059, Invitrogen) for 30 minutes at room temperature. Cell nuclei were labeled with Propidium Iodide (0.04 μg/ml) for 1 minute. Cover slips were washed with water and mounted on microscope slides with Vectashield mounting medium. Imaging was conducted on Eclipse E800 microscope (Nikon, Japan)

### Proliferation kinetics

Cells were seeded at the density of 0.5 × 10^4^ cells/ ml in 6 well plates or at the density 1.6 × 10^5^/ml in 6 cm tissue culture dishes. The medium and growth factors were changed daily and proliferation kinetics was determined by cell counting at the indicated time points using hemocytometer or TC100 Automated Cell Counter (BioRad).

### MTT assay

Primary, secondary and tertiary neurospheres were dissociated and 10^4^ cells were seeded in 100 μl of media in 96 well anti adhesive plates. 20 μl of 5 mg/ml MTT solution in PBS were added to each well and incubated for 4 hours. 100 μl of extraction buffer was added for 2 hours to dissolve the formazan crystals and the absorbance at 590 nm was assessed using Victor plate reader (Perkin Elmer).

### Neurosphere formation assay

Cells were seeded into Ultra Low Cluster Plates (Corning Life Sciences, cat. no.3471) at 5×10^4^ (SH-SY5Y; 6-well dishes) or 2×10^4^ (CHLA-15 and CHLA-20: 24-well dishes). The serum-free culture medium DMEM/F12 (Sigma) was supplemented with 60 μM putrescin, 20 nM progesterone, 5 μg/ml insulin, 100 μg/ml transferrin, 30 nM sodium selenite and 6 μg/ml glucose. Growth factors: human Epidermal Growth Factor (Gibco) and basic Fibroblast Growth Factor (Sigma) were added every 48-72 hours in final concentration of 20 ng/ ml and 10 ng/ml in media, respectively. Differentiation of primary neurospheres was obtained as described previously [31]. Neurospheres were transferred on Poly-D-Lysine coated cover-slips placed in 6 well plates and allowed to attach overnight in a drop of culture medium. To induce differentiation wells were filled with culture medium supplemented with 2% FBS. After 7-14 days the cover-slips were examined for neural differentiation and subjected to immunocytochemistry. To study secondary neurosphere formation, cells were seeded into 24 or 96-well plates at 102 cells per well. After 6-7 days the number and size of the neurospheres formed were recorded. The neurosphere diameter was assessed optically with an object micrometer.

### Western blotting

Protein samples were prepared with 4 times sample buffer (10% glycerol, 62.5 mM Tris-HCl pH 6.8, 2% SDS, 0.01 mg/mL bromophenol blue, 2% β-mercaptoethanol), and boiled for 5 minutes at 95-105 °C. Samples were loaded onto 10% polyacrylamide gels and ran at 110 volts for 4.5 hours. Proteins were then transferred to PVDF membranes through semi-wet transfer at 30 volts for 2.5 hours. Membranes were blotted in 3% milk for 2 hours to overnight. Primary antibodies were incubated overnight at 4°C (except for Actin mouse, which was incubated 1 hour at room temperature). Antibody concentrations used were as follows: Actin MAB150 1R, (Chemicon-Millipore; 1:1000), -Tubulin TU-02 (Sigma; 1:1000), CDK2 mouse D-12 (Sigma 1:1000), CDK2 rabbit M2 (Sigma; 1:500), human Spy1 (Novus; 1:1000-1:10000, Abcam; 1:1000), p27Kip1 NA35 (Calbiochem 1:100), GAP43 G9264 (Sigma 1:1000), GFAP G4546 (Sigma 1:1000), Nestin G-20 (Santa Cruz 1:1000). Membranes were washed with TBST 3 times for 5-10 minutes, followed by 1 hour incubation in secondary antibody (mouse, rabbit or goat – 1:10000) at room temperature. Membranes were then washed with TBST 3 times for 15 minutes and were visualized using FluorChemHD2imaging system (Alpha Innotech).

### Immunoprecipitation and CDK2 kinase assay

Bradford assays were performed to ensure equal protein loading, and 100-250 μg of protein was immunoprecipitated from SH-SY5Y cell lysates. Protein was incubated with 5 μL of CDK2-mouse antibody overnight at 4°C. The following day, 5 μL of protein A sepharose beads were added to the samples and incubated for 1 hour at 4°C. Samples were then washed three times with NP-40 lysis buffer at 4°C and aspirated to a final volume of 25μl. CDK2 kinase assays were performed in kinase buffer (50 mMTris-Hcl pH 7.5, 10 mM MgCl2, 1 mM DTT, 20 mM EGTA) and 0.5 μCi/μL [γ^−32^P]ATP-~3,000 Ci/mmol obtained from Perkin Elmer. 4 μg of Histone H1 was added to each 25 μl immunoprecipitated sample (described above) incubated with 25 μL of 2 times kinase buffer. Samples were incubated at 30°C for 30 minutes, followed by the addition of 25 μL of 4 times sample buffer. Samples were boiled for 5 minutes at 95-105°C and loaded onto 10% polyacrylamide gels. SDS-PAGE and transfer were performed as described above. Membranes were exposed using a Cyclone PlusPhosphoimager (Perkin Elmer), and analyzed using OptiQuant software.

### qRT-PCR

Total RNA was extracted from cells or tissues utilizing RNAeasyPlus Mini Kit (Qiagen) and reverse transcribed using 200U Superscript II (Invitrogen), 0.5 μg OligodT's and 0.5 μg random nanomers (Sigma) according to the manufacturer instructions. For each experiment the samples were reverse transcribed at the same time and cDNA was stored at −20°C. Real time PCR with SYBR green (Applied Biosystems) fluorescent detection and 200-250 nM of each primer was performed using ABI Prism 7300 or Viia7 thermocycler. Primers were designed using Primer Express software. *GAPDH* was used as the endogenous control. Data was analyzed using ABI 7300 or Viia7 software and represented as log_10_ relative quantification (RQ) or RQ relative to control.
